# Prognostic role of microRNA 182 and microRNA 18a in locally advanced triple negative breast cancer

**DOI:** 10.1371/journal.pone.0242190

**Published:** 2020-11-11

**Authors:** Rajat Bajaj, Rupal Tripathi, T. S. Sridhar, Aruna Korlimarla, Kumardeep Dutta Choudhury, Moushumi Suryavanshi, Anurag Mehta, Dinesh Chandra Doval

**Affiliations:** 1 Department of Medical Oncology, International Oncology Services, Fortis Hospital, UP, India; 2 Department of Research, Rajiv Gandhi Cancer Institute & Research Centre, Delhi, India; 3 Department of Molecular Medicine, St John’s Research Institute, Karnataka, India; 4 Department of Molecular Diagnostics and Cell Biology, Rajiv Gandhi Cancer Institute and Research Centre, New Delhi, India; 5 Department of Pathology, Rajiv Gandhi Cancer Institute & Research Centre, Delhi, India; 6 Department of Medical Oncology, Rajiv Gandhi Cancer Institute & Research Centre, Delhi, India; Fondazione IRCCS Istituto Nazionale dei Tumori, ITALY

## Abstract

**Background:**

The study assessed the epigenetic regulation and the role of microRNA (miR) expression in locally advanced triple negative breast cancers (TNBC) and comparison with the clinico-pathological variables and survival.

**Methods:**

Fifty patients of locally advanced TNBC during the period 2011–2013 were included. Expression level of test microRNA (miR-182 and miR-18a) was determined using Taqman quantitative Real time polymerase chain reaction (qRT-PCR) from formalin fixed paraffin embedded biopsy blocks. Clinical and demographic information and survival data was retrieved from the Hospital medical records.

**Results:**

An improved clinical complete response (cCR) was observed in patients with age ≥ 45 years (80%), premenopausal status (70%), tumor size < 6 cms (80%), nodal status N0-N1 (95%) and grade II-III tumor (80%). A statistically significant correlation was observed on comparison of cCR with menopausal status (p-value 0.020), T category (p-value 0.018) and the clinical nodal status (p-value 0.003). pCR also correlated with clinical nodal status (p-value 0.008). Epigenetically, miR-18a under expression (< 8.84) was most commonly associated with tumor size < 6 cms (76.7%), clinical nodal status N0-N1 (90%), cCR (60%) and pCR (53.3%). A similar trend was observed with miR-182. Statistical significance was observed with T category (p-values 0.003 and 0.004), clinical nodal status (p-values 0.001 and 0.001), clinical response (p-values 0.002 and 0.002) and pathological response (p-values 0.007 and 0.006) with respect to miR-18a and miR-182, respectively. Also, the menopausal status significantly correlated with the miR-182 expression (p-value 0.009). miR-182 overexpression (≥ 6.32) was not observed in any of the postmenopausal patients. A univariate cox proportional hazard regression model also showed statistical interactions (p-values <0.004).

**Conclusion:**

miR-182 and miR-18a overexpression correlates with worse clinical and pathological tumor characteristics in locally advanced TNBC and hence could be used to predict the outcomes and prognosis in these patients.

## Introduction

Breast cancer is the second most common cancer in the world with 2088849 new cases in 2018 and constituting around 6.6% of all cancer deaths in the world. In India, it is the most common cancer among both sexes with 162468 new cases in 2018(14% of all new cancer cases) [1]. Breast cancer has always been considered as a disease of the developed and high income countries, however, more recently, the developing countries are showing a steady rise in the number of breast cancer cases [2].

Breast cancer is a heterogeneous disease which is subdivided into different entities, each having their own clinical features and prognostic implications. A recent report from India suggests that the proportion of early breast cancer (EBC) and locally advanced breast cancer (LABC) cases are nearly equal (~51% EBC and ~49% LABC) [3]. About 10–15% of all breast cancers are estrogen receptor (ER), progesterone receptor (PR) and Her 2 Neu negative, referred to as triple negative breast cancers (TNBC). These cancers have higher histological grade and are more aggressive locally with increased chances of distant visceral metastasis [4]. The prognostic and predictive implications of ER, PR and HER 2 Neu markers in breast cancer management cannot be overemphasized. Since TNBCs lack these markers, there is a need to search for other potential biomarkers predicting the nature of the disease and prognosis in such cancers [5]. Lehmann et al revealed that TNBC can be classified into 6 different molecular subtypes with differing biological characteristics through mRNA expression [6]. The microRNA (miR) profiles of these tumors are likely to be different too [7, 8].

Reports suggest that the major determinant of tumor response to treatment is not the anatomical prognostic factors but rather the intrinsic molecular characteristics [5, 9, 10]. There has been a growing interest in breast cancer characterization based on the gene expression profiling for a better prognostication of the disease [11]. In the recent years, there has been a paradigm shift from studying about the genetics of breast cancer to exploring the epigenetic factors and this has led to the linking of the miR expression profiles with the different stages of tumor growth in terms of local spread, invasion, progression and metastasis, thus making miRs an important tumor biomarker [12]. In breast cancer, some miRs have been shown to upregulate the functions of oncogenes while others stimulate the tumor suppressors [13]. Numerous miRs, particularly miR-182 and miR-18a have been proved to be encoded in cancer-related gene regions, thereby revealing that alteration of miR expression may have a causal relationship with tumorigenesis, aggressive disease and chemo resistance [14]. Furthermore, the oncogenic properties of miR-182 and miR-18a in various tumors have been elucidated [15–19]. Previous studies demonstrated that miR-182 could regulate many suppressor genes in breast cancer, including *BRCA1*, *RECK*, *PFN1*, *FOXO1*, *ZEB1* and *HSF2* [20–24]. MicroRNA-182 (miR-182) was reported to have oncogenic potential in many cancers [25, 26]. It has also been shown that high miR-18a expression in post neoadjuvant chemotherapy residual tumors was found to be associated with a poor overall survival and a trend towards a poorer disease-free survival was observed as compared to the low miR-18a expressing post neoadjuvant chemotherapy residual tumors [27].

Over the last decade, expressions of many miRs have been implicated in the pathobiology of TNBC which includes controlling its properties of proliferation and response to chemotherapy [7]. There is a paucity of data related to the role of specific miRs in locally advanced TNBCs [7, 8]. miR-182 and miR-18a were specifically studied in the present analysis since there is extensive data in the literature about the roles of these miRs in TNBC behaviours and outcomes, however, their association with outcomes after neo-adjuvant treatment is yet to be studied. The present study was conducted to analyze and correlate the levels of these miRs (miR-182 and miR-18a) with the clinico-pathological features in locally advanced TNBC patients treated at the largest tertiary care cancer centre in North India.

## Material and methods

The present study was conducted in the Departments of Medical Oncology and Pathology at our Hospital and patients registered from January 2011 to December 2013 were recruited in the study. Medical records of these patients were reviewed retrospectively. Information related to the demographic profile, tumor type, histopathology details, neoadjuvant chemotherapy regimen, response and follow up information was recorded. The study was approved by the Institutional Review Board of Rajiv Gandhi Cancer Institute & Research Centre, Delhi, India and was conducted in accordance with the Declaration of Helsinki. All the data were fully anonymized before accessing them and the stored Formalin Fixed Paraffin Embedded (FFPE) blocks of the patients were used for this study with a waiver of informed consent.

A total of 50 patients with a diagnosis of locally advanced TNBC were included in the study. The cases were first diagnosed with invasive breast cancer via core needle biopsy and then subjected to staging work up with appropriate imaging modality to exclude the distant metastasis. All the patients included in the study had received neo-adjuvant chemotherapy with anthracyclines and taxanes, concurrently or sequentially during the period of the study. Clinical response evaluation was done in all the patients at the end of the neo-adjuvant chemotherapy with PET-CT using RECIST 1.1 criteria [28]. After recording the clinical response, the patients underwent surgery (breast conservation surgery or modified radical mastectomy) three to four weeks after the last dose of the neo-adjuvant chemotherapy. Patients who did not complete neo-adjuvant treatment (minimum 3 cycles) or did not respond to neo-adjuvant chemotherapy were excluded from the study.

FFPE blocks of the patients were cut at 4 μm thickness and the sections were mounted on Poly-L-Lysine coated slides and ER, PR and Her2 neu receptor status were studied as per the standard procedures [29]. For miR analysis, all RNA and miR extractions and cDNA conversions were performed in a few batches after the collection of the last specimen. Total RNA was extracted using reagents according to the manufacturer’s protocol (Qiagen miRNeasy Micro FFPE Kit). MiR present in the total RNA extracted was converted to cDNA using stem-loop primers specific for the chosen miR according to the published protocols. The expression levels of test genes [miR-182 (hsa-miR-182-002334) and miR-18a (PN4427975)] was determined in terms of the Ct values along with one reference genes [*RNU48* (RNU48–001006)] which was used for data normalization. TaqMan miR inventoried assays for qRT-PCR (Applied Biosystems) were used for each of the test and control miRs (RU48 and the test miRs, miR-182 and miR-18a). To determine the relative transcript abundance, Ct values of the reference miR RU48 was subtracted from that of the test miRs. This value obtained was the dCt or the delta Ct value. Normalized values were represented as relative normalized units (RNU) which were calculated and represented as 15-ΔCt. The value of 15 was chosen to encompass the dynamic range of the assay which is approximately 15 cycles from the earliest cycle to the latest cycle of reference miR, RU48. One unit increase in RNU was assumed to reflect a two-fold increase of the template [30]. Therefore, the relative normalized value (RNU) of the test miRs was “15 minus dCt”. This method was merely for the ease of the representation of the relative expression and could be variable according to the dynamic range of the assay [31]. The derived RNU values were divided into quartiles and the 3rd quartile value obtained was taken as the cut-off value for both miR-182 and miR-18a to define the high or low expression.

Receiver operating characteristic (ROC) analysis was used to evaluate the sensitivity and specificity of the miR based prognostic model in predicting outcomes. Initially, the median value of miR-18a and miR-182 distribution as the cut off was selected. However, the sensitivity and specificity were 60% and 50%, respectively. When the cut off was raised to 3Q of 7.8 and 7.4 respectively, the specificity was however raised to 78% though sensitivity was decreased. In order to achieve higher specificity, 3Q cut off [31, 32] was selected. This also reflected in the response to therapy where high expression of both miRs reflected poor response to NACT.

SPSS version 21 for Windows (SPSS Inc, Chicago IL, USA) and MedCalc version 12.5 for windows (MedCalc Software, Ostend, Belgium) were used for the statistical analysis. The descriptive statistics was done using mean or median with standard deviation (SD) or inter quartile range (IQR) for quantitative variables and categorical variables were presented in frequencies along with the respective percentages. The statistical comparisons for quantitative variables were done using unpaired t-test or Mann-Whitney ‘U’ test between the two groups and ANOVA/ Kruskal Wallis test was used to compare more than two groups. For categorical variables, Chi-square or Fisher’s exact test were used as per the nature of the data. Pearson correlation coefficient/ Spearman rank correlation coefficient was used to correlate the quantitative parameters with each other. Survival analysis was performed using the Kaplan Meier method [33]. Log Rank test was used to compare the difference in survival among the groups. A two sided p-value <0.05 was considered as significant.

## Results

A total of 50 patients with locally advanced TNBC were included in the study. The median age of the patients was 49 years. A total of 39 (78%) patients were ≥ 45 years of age and 84% patients were premenopausal. The demographic and tumor profile of the patients is shown in Table 1. The tumor was most commonly located in the upper region in 33 (66%) patients and the size was <6 cms in 60% patients. Only 30% patients had a presence of lymphovascular invasion. Grade III tumors were observed in 22% patients.

**Table 1:** Demographic and tumor profile of 50 patients with locally advanced TNBC.

Characteristics	n (%)
**Age (years)**	
< 45 years	11 (22)
≥ 45 years	39 (78)
**Menopausal status**	
Premenopausal	42 (84)
Postmenopausal	8 (16)
**Location of tumor**	
Central	11 (22)
Upper outer	23 (46)
Upper inner	10 (20)
Lower outer	5 (10)
Lower inner	1 (2)
**T category**	
< 6 cms	30 (60)
≥ 6 cms	20 (40)
**Lymphovascular invasion**	
Absent	35 (70)
Present	15 (30)
**Clinical nodal status**	
cN0	2 (4)
cN1	34 (68)
cN2	11 (22)
cN3	3 (6)
**Pathological nodal status**	
pN0	18 (36)
pN1	9 (18)
pN2	12 (24)
pN3	11 (22)
**Grade of tumor**	
I	14 (28)
II	25 (50)
III	11 (22)

A correlation of the clinical complete response (cCR) and pathological complete response (pCR) with the tumor profile and survival is given in Table 2. An improved cCR was observed in patients with age ≥ 45 years (80%), premenopausal status (70%), tumor size < 6 cms (80%), nodal status N0-N1 (95%) and grade II-III tumor (80%). In terms of cCR, a statistically significant correlation was observed on comparisons with menopausal status (p-value 0.020), T category (p-value 0.018) and the clinical nodal status (p-value 0.003). A similar trend in terms of the frequencies was observed on comparisons with pCR, however, statistical significance was only noted for the clinical nodal status (p-value 0.008).

**Table 2:** Correlation of clinical complete response and pathological complete response with the tumor profile and survival.

Characteristics	Total N = 50 n (%)	cCR N = 20 n (%)	No cCR N = 30 n (%)	p-value	pCR N = 18 n (%)	No pCR N = 32 n (%)	p-value
**Age (years)**				0.78			0.97
< 45 years	11 (22)	4 (20)	7 (23.3)		4 (22.2)	7 (21.9)	
≥ 45 years	39 (78)	16 (80)	23 (76.7)		14 (77.8)	25 (78.1)	
**Menopausal status**				**0.020**			0.082
Premenopausal	42 (84)	14 (70)	28 (93.3)		13 (72.2)	29 (90.6)	
Postmenopausal	8 (16)	6 (30)	2 (6.7)		5 (27.8)	3 (9.4)	
**T category**				**0.018**			0.054
< 6 cm	30 (60)	16 (80)	14 (46.7)		14 (77.8)	16 (50)	
≥ 6 cm	20 (40)	4 (20)	16 (53.3)		4 (22.2)	16 (50)	
**Clinical nodal status**				**0.003**			**0.008**
cN0-cN1	36 (72)	19 (95)	17 (56.7)		17 (94.4)	19 (59.4)	
cN2-cN3	14 (28)	1 (5)	13 (43.3)		1 (5.6)	13 (40.6)	
**Grade of tumor**				0.304			0.495
I	14 (28)	4 (20)	11 (33.3)		4 (22.2)	11 (34.4)	
II-III	36 (72)	16 (80)	20 (66.7)		14 (77.8)	21 (65.6)	

The levels of miRNAs in 50 TNBC patients are shown in the S1 Data. A comparison of the tumor profile and survival with the miR expression was also studied and the same has been profiled in Table 3. miR-18a under expression (<8.84) was most commonly associated with tumor size < 6 cms (76.7%), clinical nodal status N0-N1 (90%), cCR (60%) and pCR (53.3%). A similar trend was observed with miR-182 except in the case of pCR (pCR and no pCR– 50% each). Statistical significance was observed in the case of T category (p-values 0.003 and 0.004), clinical nodal status (p-values 0.001 and 0.001), clinical response (p-values 0.002 and 0.002) and pathological response (p-values 0.007 and 0.006) with respect to miR-18a and miR-182, respectively. Also, the menopausal status significantly correlated with the miR-182 expression (p-value 0.009). Interestingly, miR-182 overexpression (≥ 6.32) was not observed in any of the postmenopausal patients.

**Table 3:** Correlation of the tumor profile and survival with the miR expression.

Characteristics	Total N = 50 n (%)	miR-18a Over expression (≥ 8.84) N = 20 n (%)	miR-18a Under expression (< 8.84) N = 30 n (%)	p- value	miR-182 Over expression (≥ 6.32) N = 18 n (%)	miR-182 Under expression (< 6.32) N = 32 n (%)	p- value
**Age (years)**				0.71			0.92
< 45 years	11 (22)	4 (20)	7 (23.3)		3 (16.7)	8 (25)	
≥ 45 years	39 (78)	16 (80)	23 (76.7)		15 (83.3)	24 (75)	
**Menopausal status**				0.131			**0.009**
Premenopausal	42 (84)	18 (90)	24 (80)		18 (100)	24 (75)	
Postmenopausal	8 (16)	2 (10)	6 (20)		0 (0)	8 (25)	
**T category**				**0.003**			**0.004**
< 6 cms	30 (60)	7 (35)	23 (76.7)		6 (33.3)	24 (75)	
≥ 6 cms	20 (40)	13 (65)	7 (23.3)		12 (66.7)	8 (25)	
**Clinical nodal status**				**0.001**			**0.001**
cN0-cN1	36 (72)	9 (45)	27 (90)		8 (44.4)	28 (87.5)	
cN2-cN3	14 (28)	11 (55)	3 (10)		10 (55.6)	4 (12.5)	
**Grade of tumor**				0.700			0.376
I	14 (28)	5 (25)	9 (30)		4 (22.2)	10 (31.3)	
II-III	36 (72)	15 (75)	21 (70)		14 (77.8)	22 (68.7)	
**Clinical response**				**0.002**			**0.002**
cCR	20 (40)	2 (10)	18 (60)		2 (11.1)	18 (56.3)	
No cCR	30 (60)	18 (90)	12 (40)		16 (88.9)	14 (43.7)	
**Pathological response**				**0.007**			**0.006**
pCR	18 (36)	2 (10)	16 (53.3)		2 (11.1)	16 (50)	
No pCR	32 (64)	18 (90)	14 (46.7)		16 (88.9)	16 (50)	

A univariate cox proportional hazard regression model was also developed to see the combined effect of the parameters including clinical N status, grade, presence of lymphovascular invasion and miR expression and survival time (Table 4). Statistical significance was observed for all these parameters (p-values <0.004) except with the grade of the tumor.

**Table 4:** Univariate cox proportional hazard regression.

Characteristics	Odds ratio	95% CI	p-value
**Clinical nodal status**		1.417–5.602	**0.003**
Low nodal status	1		
High nodal status	2.817		
**Grade**		0.650–2.780	0.425
Low grade	1		
High grade	1.344		
**Presence of LVI**		1.917–7.545	**0.0001**
No	1		
Yes	3.803		
**miR-18a expression**		2.021–7.574	**0.0001**
Under expression	1		
Over expression	3.913		
**miR-182 expression **		2.053–8.484	**0.0001**
Under expression	1		
Over expression	4.174		

CI, confidence interval; N, node; LVI, lymphovascular invasion.

Multivariate Cox regression analysis was also performed and nodal positivity had a hazard ratio of 14.102 (95%CI 2.934–67.774, p-value 0.001) with respect to miR-18a while it was 11.929 (95%CI 2.238–63.579, p-value 0.004) for miR-182. The HR for all the other parameters including tumor size, age group and stage group for both miR-18a and miR-182 was in the range of 0.597–1.385 and statistically not significant.

**Fig 1:**
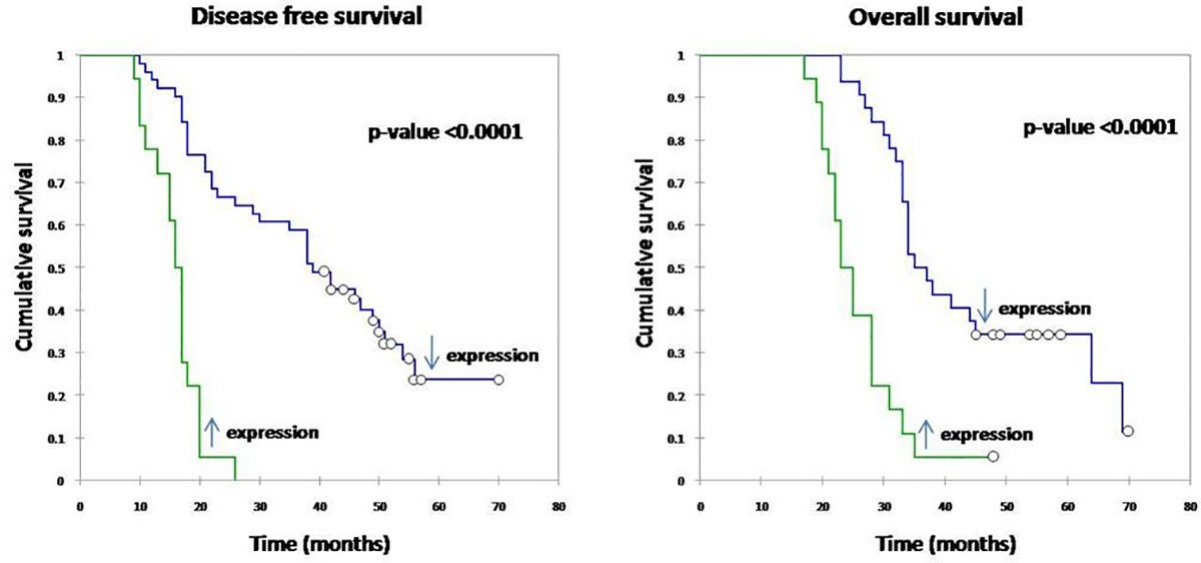
Survival analysis of patients by Kaplan Meier method based on their microRNA expression (a) Disease free survival (DFS) (b) Overall survival (OS). Underexpression of miR levels (miR-18a and miR-182) was associated with improved DFS and OS (p-value <0.0001).

The survival analysis of the patients was performed based on their miR expression levels and both disease free survival (DFS) (Fig 1A) and overall survival (OS) (Fig 1B) were studied. Expression of miR levels (miR-18a and miR-182) were significantly associated with the survival rates (p-values <0.0001). Underexpression of miR was associated with improved survival (both DFS and OS). We also analyzed the pattern of recurrence in these 50 cases of locally advanced TNBC and observed that 34 out of 50 patients had recurrence within the first two years of diagnosis, despite receiving complete treatment.

**Fig 2:**
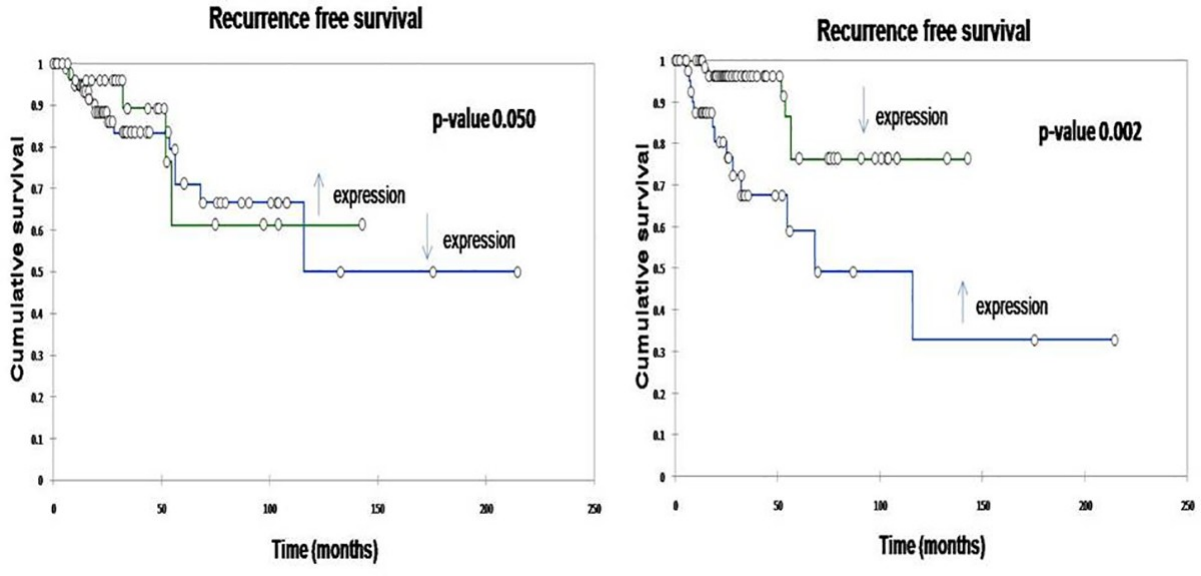
Recurrence free survival (RFS) of patients by Kaplan Meier method based on their microRNA expression (a) miR-18a (b) miR-182. Overexpression of miR-18a was associated with improved RFS (p-value 0.050). Underexpression of miR-182 was associated with improved RFS (p-value 0.002).

To confirm the relevance of miR-182-5p in an independent cohort of human breast cancers, we compared the data with that from TCGA (The Cancer Genome Atlas) from the breast cancer database, of which, TNBC comprised of 120 cases. The relative abundance of miRs was counted as the reads per million of miR-182-5p across the tumors where we initially found a significant difference (P < 0.0001) between estrogen receptor positive tumors and TNBC in terms of its level of expression. We followed a similar method of third quartile CO and survival analysis was performed between both miR-182 and miR-18a as high and low expression groups. miR-182 acted as a clear independent predictor (Confirmed by COX analysis) with high miR-182 expression showing poor survival with p value<0.01. miR-18a showed a similar trend although it did not reach the statistical significance (Fig 2).

## Discussion

Breast cancer accounts for around 30% of all cancers in females and LABC represents the most aggressive breast cancer subtype [34]. Till date, there are no published reports which establish the concrete role of specific miRs in the unique cohort of locally advanced TNBCs. The present study was therefore conducted to analyze and correlate the levels of miRs with the clinico-pathological features in a cohort of 50 patients with locally advanced (stage III) TNBC treated with neoadjuvant chemotherapy at one of the largest tertiary care cancer centres in North India.

In our study, cCR was observed in 20/50 patients (40%) post neoadjuvant chemotherapy which comprised of both taxanes and anthracyclines. Carey et al [35] in their study on TNBC patients had reported lower cCR (29%), however, the patients had received only neoadjuvant anthracyclines in that subset. Taxanes were not administered to all the patients which could have led to a lower cCR in their study. In our study, we observed a good concordance between cCR by MRI/PET-CT after neoadjuvant chemotherapy and pCR in the surgical specimens with 18/20 (90%) patients who had cCR also having pCR. Chang et al and Bear et al in their studies showed that cCR did not correlate very well with the pCR rates [36, 37], but both these studies evaluated both the hormone positive and negative breast cancers which could explain the finding. pCR was attained in 18/50 (36%) patients in our study. This is consistent with the previous studies which have found high pCR rates in TNBC patients ranging from 38% to 45% [35, 38–40]. There are many recent studies which have shown very high rates of pCR upto 60% by adding platinums or capecitabine to the neoadjuvant taxanes and anthracyclines [41, 42]. While this approach needs to be further validated and such robust pCR rates need to be correlated with survival, it certainly looks promising. In the present study, platinum was not used in the patients because this study recruited patients in a specific time frame, presenting to our Hospital during 2011 to 2013. We defined pCR as absence of residual invasive tumor in breast or axilla (ypT0-is N0 status). Wide range of pCR rates of 13–65% have been reported in various studies [43–45]. Such a huge variation in pCR rates can be attributed to factors like definition of pCR, selection of the breast cancer subtypes with high proliferation and chemo-sensitivity.

Various biological, clinico-pathologic and treatment related factors were evaluated in these studies which correlated with the clinical and pathological complete response. We found that achievement of cCR was significantly more in patients with less than 6 cm initial tumor size, however, the lesser tumor size did not correlate significantly with achievement of pCR. Wang et al [40] also found a positive relation between the attainment of pCR and less tumor size but this was not corroborated by Chang et al [36] in their study. It was also seen that grade of the tumor was not helpful in prediciting pCR to neoadjuvant therapy in our study. We have similar pCR rates in low grade and high grade tumors. This is in contrast to some of the other studies including one of the largest available pooled analysis related to the response to neoadjuvant chemotherapy which have reported increased pCR rates in high grade tumors [37, 46–48].

miR-182, a member of the miR-183 family located on 7q31-34 is a relatively novel miR containing a 24-bp sequence. It has been found to be involved in the regulation of many cancers like colon, ovarian and lung cancer, resulting in tumor proliferation and apoptosis, thus behaving like an oncogene [49]. Many genetic regulations have been postulated as a cause of its oncogenic function [22, 50]. It is also involved in the regulation of DNA repair gene *BRCA1* [29]. miR-182 has been implicated as a promoter in the processes including oncogenesis, growth, invasion and metastasis in breast cancer [51]. We found that high miR-182 and miR-18a expressions did not correlate with good pathologic response in TNBC patients which is in line with the observation made by Kolacinska et al in their study [52]. One reason that can be postulated for this observation is that high miR-18a imparts the property of resistance to taxanes to TNBC tumors, as was observed by Shy LY et al in their paper [53]. We also found that patients with high miR-182 expression had higher pathological lymph nodes than the other patients which were also observed by Medimegh et al in their study [54]. It was seen that high miR-182 and miR-18a expression was significantly related to poorer survival.

We have done an analysis of tumors taken from TCGA using miR expression data, though we cannot comment about the response to treatment as these were primary tumors. These results confirm that miR-182 and miR-18a are important miRs relevant to human cancer and further study is warranted to clearly delineate its role in the pathogenesis and progression in specific breast cancer subtypes.

Clinical significance of this data is of paramount importance. As our results indicate, underexpression of miR-18a has been seen to be associated with higher chances of getting a cCR and pCR in a triple negative breast cancer patient post surgery. Also, the underexpression of miR-182 correlated with the cCR observed in these patients. The miR-182 and miR-18a explored in our study can also be used in developing marked assays for TNBC patients, wherein, the expression levels of these miRs may predict the pCR.

Inherent biases may be present due to the retrospective nature of the study. To our knowledge, this is the first study which has analyzed the survival outcomes related to the epigenetic regulation with respect to the test miRs. Further studies in future are required to validate this correlation because these miRs, when used as a signature assay, could then be used to predict the outcomes and prognosis in patients with TNBC.

## Supporting information

S1 Data (XLSX)
